# Association between celiac disease and chronic hepatitis C

**Published:** 2016

**Authors:** Giovanni Casella, Davide Viganò, Carlo Romano Settanni, Olivia Morelli, Vincenzo Villanacci, Vittorio Baldini, Gabrio Bassotti

**Affiliations:** 1 *Medical Department, Desio Hospital, Desio (Monza e Brianza), Italy*; 2 *Gastroenterology and Hepatology Section, Department of Medicine, University of Perugia Medical School, Perugia, Italy *; 3 *Institute of Pathology, Spedali Civili di Brescia, Brescia, Italy *

**Keywords:** Celiac disease, Chronic hepatitis, HCV virus, Interferon therapy, Liver disease

## Abstract

Celiac disease is characterized by a gluten-induced damage of the small bowel in sensitive individuals that may cause malabsorption. Non-intestinal inflammatory diseases may trigger immunologic gluten intolerance in susceptible people and the HCV virus may be considered as a suitable candidate. Interferon therapy could precipitate symptom onset in subjects with silent celiac disease. In fact, symptoms such as diarrhea, anemia, and weight loss may occur during interferon therapy and are associated with serological positivity of anti-tranglutaminase antibodies. To date, considering the available literature data, it is very difficult to support a firm association between HCV chronic hepatitis and celiac disease. Thus, such a serological screening in HCV patients before starting interferon therapy should not be recommended. However, serology for celiac disease must be considered in patients who develop diarrhea and/or weight loss during such therapy.

## Introduction

 Celiac disease (CD) is a chronic pathological condition, with a prevalence in the Western countries of about 1/100 (1). The CD is characterized by a gluten-induced more or less severe immune-mediated damage of the small bowel mucosa in genetically predisposed individuals (2), which in the time course may lead to malabsorption (3). The CD has a wide spectrum of gastrointestinal and extraintestinal manifestations (4) and, considering the strong genetic component, may be associated with several other diseases, such as type 1 diabetes mellitus, and autoimmune thyroiditis. Of interest, hepatitis C virus (HCV) may trigger immunologic gluten intolerance in predisposed people (5) and this immunopathological basis has been considered as a possible explanation for the higher prevalence of CD in HCV positive patients (6). Moreover, therapeutic interventions aimed at controlling HCV infection, such as interferon (IFN) alpha therapy, alone or in combination with ribavirin, may enhance immunologic Th1 and Th2 lymphocytes responses that, in turn, might cause an immune mediated damage of the gut mucosa (7). 

## Possible pathogenic mechanisms

It has been hypothesized that non-intestinal inflammatory diseases may trigger immunologic gluten intolerance in susceptible people. The HCV virus may be considered as a suitable candidate, since HCV is also associated with the pathogenesis of secondary autoimmune disorders as mixed cryoglobulinemia, lichen planus, and Sjogren’s syndrome (6). The cell-mediated inflammatory response to HCV may involve T cells restricted to HLA-DQ2 (8), the class II HLA allele linked to CD and other autoimmune diseases (9). Other authors hypothesized that some viruses, including HCV, may have amino-acid sequences homologous to some gliadin epitopes; these sequences are those mainly responsible for mucosal toxicity. Thus, viral infections may act as “triggers” activating autoimmune mechanisms involved in CD development (10). This immunopathogenic pathway might also explain the possible association between CD and HCV chronic hepatitis, since a high prevalence of the HLA DQ2 haplotype, typically associated with CD (11) was found in HCV patients (6). Moreover, IFN therapy may induce or worsen autoimmune disorders and precipitate symptom onset in subjects with silent CD. In fact, symptoms such as diarrhea, anemia with hypoferritinemia, and weight loss may occur during this therapy, with the concomitant appearance of anti-tissue transglutaminase (tTG) (7). Patients with a previous diagnosis of CD, and also those on a gluten freed diet, have experienced these symptoms during IFN therapy, (12). It is also important to consider blood transfusions as a possible route of HCV transmission in celiac patients, because iron deficiency anaemia (sometimes severe) may be the only clinical manifestation of CD (13).

Routine blood screening by means of anti tTG may be questioned, because a high rate of anti tTG positivity has been reported in liver disease, particularly during liver decompensation (14). These data suggest that such a screening may not be appropriate to detect the presence of the CD in HCV chronic hepatitis. On the other hand, CD screening using anti-endomysial antibodies (EMA) is an expensive, time requiring procedure, with the bias of undergoing a subjective interpretation of the immunofluorescence pattern and the need for animal or human tissues as substrate (5).

## Clinical experience

Table 1 summarizes the available studies showing the prevalence of CD in HCV patients. 

Low prevalence studies. Thevenot et al, in their study, did not find any relationship between CD and chronic hepatitis C (5). The authors studied 624 HCV positive patients in whom the presence of IgA EMA, and IgA and IgG anti gliadin antibodies (AGA) were evaluated; IgA EMA were found in 0.16% of patients, IgA AGA in 5.7% and IgG AGA in 4.4%. Twenty-five patients underwent upper gastrointestinal endoscopy with duodenal biopsies but nobody had pathological increased CD3+ intra-epithelial lymphocytes; however, these data have been explained by the low prevalence of CD observed in France (15). Villalta et al evaluated the prevalence of IgA and IgG anti tTG in 47 autoimmune hepatitis patients, 120 blood donors and 100 chronic hepatitis C cases, and found only one HCV patient positive for IgG anti-tTG, but negative for IgA EMA (16); however, this case was considered as a false positive because HLA DQ2-DQ8 typing was negative. Gravina, et al. reported a series of 210 patients with histologically documented chronic hepatitis C, in whom the research of anti-EMA and anti-tTG antibodies revealed no evidence of positive serology for CD (17). Germenis, et al. reported a 0.54% prevalence of positive CD serology among 738 patients with different liver disease, not different than that found in healthy controls (18); the authors were unable to find any statistically proven difference between the prevalence of CD in HCV patients and healthy controls or patients with autoimmune liver disease. 

**Table 1:** Summary of the studies investigating the prevalence of CD in HCV patients

Authors	Country	HCV patients (n)	Celiac Patients (n)
Thevenot, et al	France	624	0 (0%)
Fine, et al	U.S.A	259	3 (1.2%)
Durante-Mangoni, et al	Italy	534	7 (1.3%)
Villalta, et al	Italy	100	0 (0 %)
Gravina, et al	Italy	210	0 (0%)
Ruggeri, et al	Italy	244	3 (1.2%)
Hernandez, et al	U.S.A	195	0 (0%)

## Higher prevalence studies

Fine et al reported that 1.2% (3/259) of their HCV positive patients had CD (6). Ruggeri et al discovered 5 EMA and anti-tTG IgA positive subjects out of 244 HCV patients (2%), a prevalence higher than the one found in healthy controls (0.16%) and in non-HCV patients (0.8%); in 3 of these 5 patients CD was confirmed histologically, showing a higher prevalence of disease in HCV patients (1.22%) than in a general adult population (0.18%) (19). Durante-Mangoni et al observed a higher incidence of pre-treatment IgA anti-tTG (1.3%) in a cohort of 534 HCV treated patients compared to a control-group of 225 HCV negative patients (0-4%) (7). The higher number of CD patients identified in this study might be due to an increased awareness toward this condition of the physicians involved in that geographical area (20).

Concerning the presence of HCV infection in CD patients, Silano et al found a low serologic HCV positivity (0.91%) among individuals affected by CD (21); the study of Gravina et al (17) showed a prevalence of 1.54% of HCV infection in 194 celiac patients, similar to that reported in the Southern Italy population. In the study of Hernandez et al HCV positivity was found in 6 of 878 CD patients (prevalence 0.68%) (22) and these CD patients had been diagnosed for complaints of diarrhea, weight loss, iron deficiency anaemia, and depression. Teml and Vogelsang found a prevalence of 0.6% (3/488) of HCV infection in CD patients who had risk factors such as i.v. drug abuse or blood transfusions (23). 

## Appearance of celiac disease during interferon therapy

A number of cases of clinically overt CD have been described in patients with HCV-related chronic hepatitis during treatment with IFN alpha (24-30); however, only in about half of these case reports (with no clinical or biochemical signs of malabsorption) EMA and tTG antibodies were evaluated before starting IFN therapy.

Since it has been claimed that IFN alpha therapy may trigger the development of CD in susceptible individuals (22), a serological screening for CD before starting IFN treatment has been suggested by some authors (31). Hernandez et al (22) evaluated the prevalence of CD in 194 patients with chronic hepatitis C, with 16% of them receiving IFN therapy; two HCV patients (1%) were tTG IgA positive, but EMA negative. Duodenal biopsies in these two patients were normal. Ruggeri et al (19) demonstrated that none of 42 HCV infected patients were treated with IFN alpha developed positive CD serology during the treatment, and in the study of Gravina et al (17) of 130 HCV patients were treated with IFN alpha and ribavirin no patient developed CD during and after the treatment. Durante-Mangoni et al (7) stated that the prevalence of anti-tTG IgA in HCV patients treated with IFN was 24% in those with CD symptoms, while it was 0.6% in others; furthermore, all IgA positive patients in their study had a histologically proven CD, so they hypothesized that INF therapy could trigger CD symptoms in silent patients, and that those symptoms were related to anti-tTG IgA positivity before treatment. At present, the question whether IFN alpha treatment may induce blood EMA and tTG positivity remains unanswered.

## Conclusion 

According to previous findings, it is difficult to support an association between HCV chronic hepatitis and CD. Although, liver function may be involved in a subgroup of CD patients (32,33), the possibility of coexisting liver disease should only be suspected in the presence of hypertransaminasemia when a diagnosis of CD is made, and when there is the lack of normalization of liver enzymes after one year of gluten-free diet (34). Thus, serological screening for CD in HCV patients before IFN therapy should not be recommended as a routine practice, but it should be considered in patients who develop diarrhea and/or weight loss during IFN alpha-ribavirin therapy (22), which may also lead to discontinuation of HCV treatment.
